# Identification of PPARgamma Partial Agonists of Natural Origin (II): In Silico Prediction in Natural Extracts with Known Antidiabetic Activity

**DOI:** 10.1371/journal.pone.0055889

**Published:** 2013-02-06

**Authors:** Laura Guasch, Esther Sala, Miquel Mulero, Cristina Valls, Maria Josepa Salvadó, Gerard Pujadas, Santiago Garcia-Vallvé

**Affiliations:** 1 Grup de Recerca en Nutrigenòmica, Departament de Bioquímica i Biotecnologia, Universitat Rovira i Virgili (URV), Tarragona, Catalonia, Spain; 2 Centre Tecnològic de Nutrició i Salut (CTNS), TECNIO, CEICS, Reus, Catalonia, Spain; University of South Florida College of Medicine, United States of America

## Abstract

**Background:**

Natural extracts have played an important role in the prevention and treatment of diseases and are important sources for drug discovery. However, to be effectively used in these processes, natural extracts must be characterized through the identification of their active compounds and their modes of action.

**Methodology/Principal Findings:**

From an initial set of 29,779 natural products that are annotated with their natural source and using a previously developed virtual screening procedure (carefully validated experimentally), we have predicted as potential peroxisome proliferators-activated receptor gamma (PPARγ) partial agonists 12 molecules from 11 extracts known to have antidiabetic activity. Six of these molecules are similar to molecules with described antidiabetic activity but whose mechanism of action is unknown. Therefore, it is plausible that these 12 molecules could be the bioactive molecules responsible, at least in part, for the antidiabetic activity of the extracts containing them. In addition, we have also identified as potential PPARγ partial agonists 10 molecules from 16 plants with undescribed antidiabetic activity but that are related (*i.e.*, they are from the same genus) to plants with known antidiabetic properties. None of the 22 molecules that we predict as PPARγ partial agonists show chemical similarity with a group of 211 known PPARγ partial agonists obtained from the literature.

**Conclusions/Significance:**

Our results provide a new hypothesis about the active molecules of natural extracts with antidiabetic properties and their mode of action. We also suggest plants with undescribed antidiabetic activity that may contain PPARγ partial agonists. These plants represent a new source of potential antidiabetic extracts. Consequently, our work opens the door to the discovery of new antidiabetic extracts and molecules that can be of use, for instance, in the design of new antidiabetic drugs or functional foods focused towards the prevention/treatment of type 2 Diabetes Mellitus.

## Introduction

Since ancient times, natural products (NPs) have played an important role in the treatment of type 2 diabetes mellitus (T2DM) [1]. Plants are one of the most important sources of antidiabetic compounds. Thus, 656 species from 437 genera, representing 111 plant families, with antidiabetic properties have been identified [1]. The plant families most studied as a result of their confirmed antidiabetic effects include *Leguminoseae*, *Lamiaceae*, *Liliaceae*, *Cucurbitaceae*, *Asteraceae*, *Moraceae*, *Rosaceae*, *Euphorbiaceae* and *Araliaceae* [2].

Although plant extracts have been used for the treatment of T2DM for hundreds of years in India [3], [4], China and other parts of the world, more research is needed for the identification of their active compounds and their mode of action. Some of the active principles associated with the antidiabetic activity of plant extracts are alkaloids, saponins, xanthones, flavonoids and nonstarch polysaccharides [1]. Despite the wide array of these active principles with a demonstrated antidiabetic activity, to date, metformin is the only drug approved for treatment of T2DM derived from a medicinal plant [5]. Therefore, the identification of the active compounds and the modes of action from plants traditionally used in the treatment of T2DM is an important issue for the discovery of new antidiabetic drugs and for the validation, standardization and rational use of traditional herbal remedies [1].

Numerous mechanisms of antidiabetic action have been proposed for several plant extracts [1], [6] and some hypotheses relate their effects to the increase of the insulin-stimulated glucose uptake. One target of interest for antidiabetic drugs is peroxisome proliferators-activated receptor gamma (PPARγ). PPARγ is a member of the nuclear receptor superfamily that regulate the gene expression of proteins involved in the control of glucose and lipid metabolism [7]. Indeed, the importance of PPARγ in regulating the insulin sensitivity has motivated research groups in both academia and the pharmaceutical industry to devote increasing efforts toward developing synthetic PPARγ agonists, which could be of therapeutic use in patients affected by T2DM [8]. Thiazolidinediones (TZDs) are one important class of synthetic agonists of PPARγ. TZDs are antidiabetic agents currently used in the treatment of T2DM that target adipose tissue and improve insulin sensitivity. Despite the clinical benefit of these drugs, the use of TZDs has been associated with adverse effects including weight gain, increased adipogenesis, renal fluid retention and possible increased incidence of cardiovascular events [9], [10]. Therefore, new PPARγ ligands with enhanced therapeutic efficacy and reduced adverse effects are needed. A promising new class of such ligands is selective PPARγ modulators (*i.e.*, SPPARγMs) [9], [10]. These compounds act as partial agonists of PPARγ and display different binding properties in comparison to full agonists [11], [12]. Several natural products or plant extracts have been found to increase insulin-stimulated glucose uptake through the action of PPARγ with no or little effect on adipocyte differentiation [6], [13]–[15]. Thus, PPARγ partial agonists from natural extracts are promising candidates for the treatment of T2DM. There are successful examples of the application of structure-based drug design methods to discover new PPARγ partial agonists from natural products [16], [17].

Based on the hypothesis that it would be possible to identify PPARγ partial agonists among medicinal extracts previously used as hypoglycemic agents, the goal of the present work was to find natural extracts with known antidiabetic activity that contain at least one molecule that we predict as a PPARγ partial agonist through a virtual screening (VS) workflow that has previously been carefully validated experimentally [18]. Our results provide new information about potential active molecules of natural extracts with antidiabetic properties and their mode of action, *i.e.*, the increase of the insulin-stimulated glucose uptake through the action of PPARγ. We also suggest plants with undescribed antidiabetic activity that may contain PPARγ partial agonists and are related to plants with known antidiabetic activity. These plants represent a potential new source of antidiabetic extracts. In addition, the new PPARγ partial agonists that we have predicted are chemically different from known PPARγ partial agonists and could be used as lead-hopping candidates for the development of new antidiabetic drugs.

## Results and Discussion

### Virtual Screening Description, Validation and Application

We used a slightly modified version of a VS workflow that was previously developed and validated experimentally [18] to identify PPARγ partial agonists from a large in-house database of compounds. Briefly, the VS used consists of a combination of two pharmacophore modeling methods (*i.e.*, one of them to discard potential PPARγ full agonists and the second one to identify PPARγ partial agonists), a protein-ligand docking and an electrostatic and shape similarity search. The discriminatory power of the VS workflow to identify PPARγ partial agonists was evaluated by applying it to a group of 211 known PPARγ partial agonists obtained from the literature and to 3,122 decoys obtained from the DUD database [19]. See Table 1 for data about how many of these molecules *survived* each VS step. Because we were interested in discovering novel PPARγ partial agonists but not full agonists, we developed an initial structure-based pharmacophore, called the *antipharmacophore*, to exclude possible full agonists. We used this strategy because full agonists present more clearly defined features than partial agonists. Although both types of agonists interact with the ligand-binding domain of PPARγ through several hydrophobic contacts, their mode of binding, and thus their effects, are different [11], [12]. Full agonists are characterized by making a hydrogen-bond network with Ser289, Tyr473, His323 and His449 PPARγ residues, but most partial agonists form a hydrogen bond with Ser342 [11]. In total, 135 known PPARγ partial agonists and 2,204 decoys survived the antipharmacophore step, *i.e.*, they were not identified as potential PPARγ full agonists and served as the input molecules in the next step (Table 1). From the molecules that survived the antipharmacophore step, 111 known PPARγ partial agonists and 964 decoys were identified as PPARγ partial agonists by our partial agonist pharmacophore. This represents an enrichment factor (**EF**) of 1.79 (Table 1). To find docking poses that were compatible with the partial agonist pharmacophore, the compounds that had at least one conformer, generated *in vacuo*, that matched with the partial agonist pharmacophore were also docked to the PPARγ structure from 2Q5S. The best docking poses were then matched again to the partial agonist pharmacophore, identifying that 72 out of 111 partial agonists and 382 out 964 decoys that *survived* the previous step have at least one docked pose that simultaneously accomplished the following: (a) compatibility with the PPARγ ligand-binding site; and (b) possession of functional groups that match the 3D location of the sites of the partial agonists pharmacophore. Finally an electrostatic and shape similarity analysis was applied. Using the experimental poses of five known PPARγ partial agonists as queries, 65 out of 72 partial agonists and 102 out 382 decoys were identified as partial agonist candidates by this VS step (Table 1). In terms of sensitivity (**Se**), specificity (**Sp**) and **EF**, the electrostatic/shape similarity analysis was the best step of the VS (Table 1). Overall, our VS workflow identified as partial agonists 65 and 102 out of the initial 211 and 3,122 molecules labeled as partial agonists and decoys, respectively. Therefore, the **EF** of the process was 6.15 (a 38.92% of 15.80 that would correspond to the highest possible **EF** value) and the **Se** and the **Sp** were 30.81% and 96.73%, respectively. The high **Sp** and moderate **Se** of our procedure reflect the correct assignment of inactive compounds and the loss of potential partial agonists, respectively. However, because of the high number of initial compounds and the difficulties in differentiating partial from full agonists, we preferred a specific, but less sensible, VS workflow. This VS workflow therefore seems adequate to identify molecules with antidiabetic properties that could act as PPARγ partial agonists.

**Table 1:** Validation and application of the Virtual Screening (VS) workflow.

Set of Compounds	Initial Number of Compounds	Structure-based pharmacophore screening			Electrostatic/shape similarity analysis
		anti pharmacophore	partial agonist pharmacophore		
		*in vacuo conformations*	*in vacuo conformations*	*docking poses*	
Partial Agonists	211	135	111	72	65
Decoys	3,122	2,204	964	382	102
EF			1.79	1.54	2.45
Sensitivity (Se)			82.22%	64.86%	90.28%
Specificity (Sp)			56.26%	60.37%	73.3%
NP database	29,779	21,705	2,899	935	65

A dataset of 211 known PPARγ partial agonists and 3,122 decoys extracted from the DUD database were used to validate our VS workflow. Once the VS was validated, it was applied to a dataset of 29,779 natural products (NPs). The numbers represent the number of compounds from each set that *survived* each step when applied sequentially.

Once the VS workflow was validated, it was applied to an in-house database formed by 29,779 NPs that contained an annotation of their natural source. After applying the VS workflow described above, a group of 65 PPARγ partial agonist candidates were ultimately identified (see Table 1 for viewing the number of molecules that survived each step of the VS workflow).

### Virtual Screening Hits in Natural Extracts with Known Antidiabetic Activity

**Figure 1:**
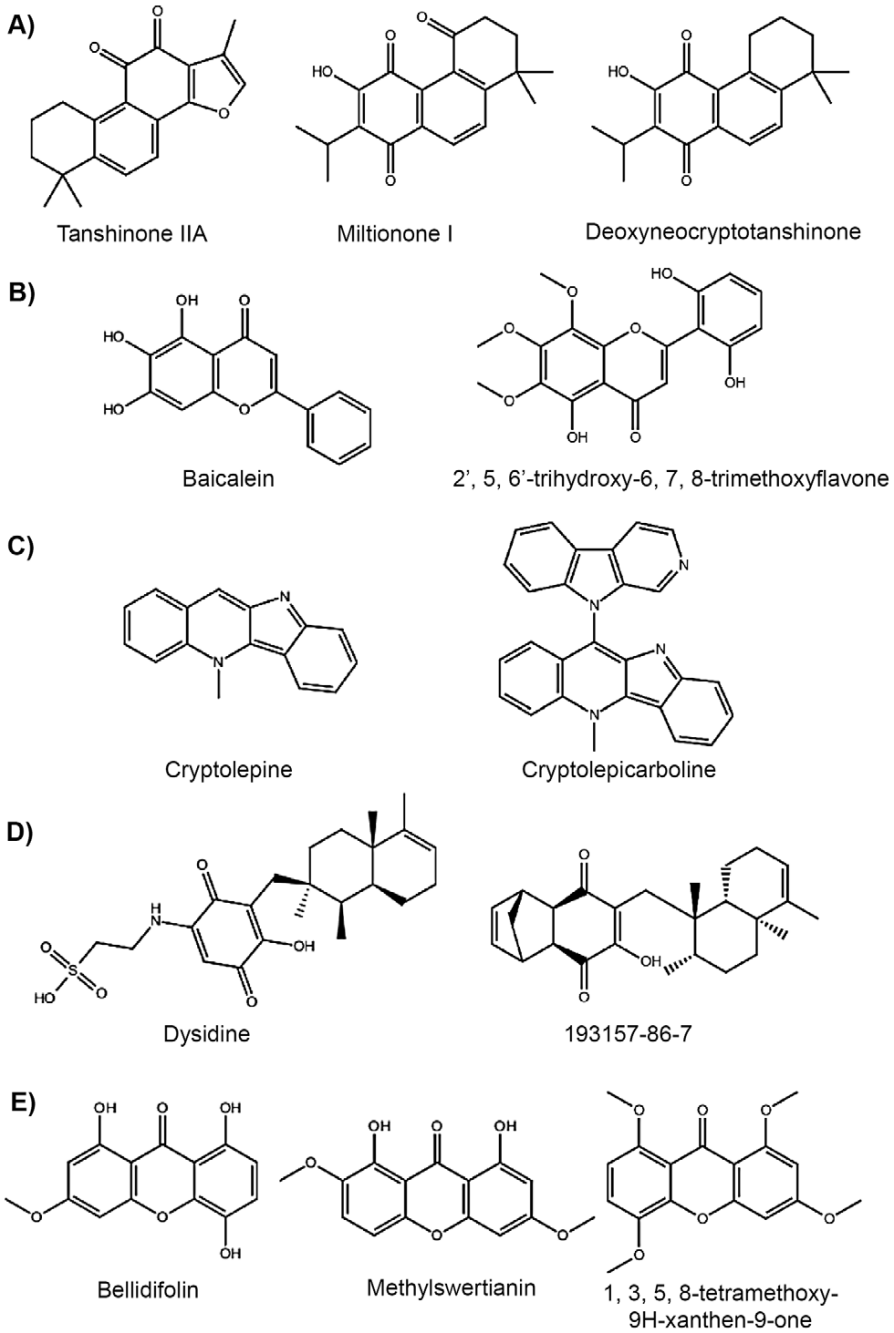
Chemical comparison between molecules that we predict as PPARγ partial agonists and molecules with described antidiabetic activity. Each row represents the comparison of the 2D chemical structure between a molecule predicted as a PPARγ partial agonist through our VS workflow and a similar molecule that has been described to present antidiabetic activity.

According to the information available in our in-house NP database, the 65 molecules that were predicted by the VS workflow as potential PPARγ partial agonists have been isolated from 74 different natural sources. Interestingly, a systematic bibliographic search of PubMed (http://www.pubmed.org) revealed that 11 out of these 74 natural extracts were described previously as having antidiabetic activity (Table 2). These 11 extracts contained 12 molecules that we predict to be PPARγ partial agonists (see Table 2 and Figure S1), therefore, it is plausible that they could contribute to the observed antidiabetic activity of their corresponding extracts. In fact, a search with SciFinder (http://www.cas.org/products/sfacad) revealed that 6 out of these 12 natural compounds are extremely similar to molecules for which antidiabetic properties have already been described (Table 2 and Figure 1), although no mechanism of action has been suggested for them. This finding validates our methodology and suggests that the mode of action of these molecules could be through PPARγ. The remaining 6 natural compounds not identified previously as antidiabetic molecules represent new molecules with this activity. The most significant compounds found in these 11 antidiabetic extracts will be discussed below:

**Table 2:** Natural extracts with described antidiabetic activity that contain one molecule that is predicted to be a PPARγ partial agonist by our virtual screening protocol.

Molecule Name (CAS number)	Cluster	Extract	Kingdom - Family	Ref. Isolation Molecule from Extract	Ref. Antidiabetic Extract	Ref. Antidiabetic Molecule
7-hydroxy-3,5,8-trimethoxyflavone (71706-62-2)	8	*Achyrocline satureoides*	Plantae - Asteraceae	[49]	[50]	-
5-hydroxy-7,8,2′,3′-tetramethoxyflavone (4767-67-3)	8	*Andrographis paniculata*	Plantae - Acanthaceae	[51], [52]	[53]	-
xanthoangelol F (265652-71-9)	7	*Angelica keiskei*	Plantae - Apiaceae	[54], [55]	[30]	[30]
3-[4-(1H-indol-3-yl)-2,3,5,6-tetramethoxyphenyl]-7-(3-methyl-2-buten-1-yl)-1H-indole (78279-81-9)	7	*Aspergillus terreus*	Fungi - Trichocomaceae	[56]	[57]	-
Cryptolepicarboline (171090-86-1)	24	*Cryptolepis sanguinolenta*	Plantea - Apocynaceae	[27], [58]	[28]	[28]
(193157-86-7)	1	*Dysidea villosa*	Animalia - Dysideidea	[59]	[29]	[29]
2,4,6,2′,6′-pentamethoxybiphenyl (93236-65-8)	8	*Fucus vesiculosus*	Chromalveolata -Fucaceae	[60]	[61]	-
bazouanthrone (942983-94-0)	3	*Harungana madagascariensis*	Plantae - Hypericaceae	[62]	[63]	-
hericerin (140381-53-9)	7	*Hericium erinaceum*	Fungi - Hericiaceae	[64]	[65]	-
deoxyneocryptotanshinone (27468-20-8)	2	*Salvia miltiorrhiza*	Plantae -Lamiaceae	[66]	[21]	[24]
miltionone I (125675-06-1)	2	*Salvia miltiorrhiza*	Plantae - Lamiaceae	[67]	[21]	[24]
2′,5,6′-trihydroxy-6,7,8-trimethoxyflavone (98187-98-5)	8	*Scutellaria baicalensis*	Plantae - Lamiaceae	[68]	[25]	[69]

The Table shows the natural extracts (*i.e.*, third column) and the VS hits that have been purified from them (identified by their common name, when available, and CAS number). The bibliographic references for each extract are split in three columns where (a) the fifth column reports papers that describe the purification of each molecule from the corresponding extract; (b) the sixth column reports papers that describe the antidiabetic activity of the corresponding extract; and (c) the seventh column reports papers that describe the antidiabetic activity of the corresponding molecule or similar molecules (when available). The second column represents the number of the cluster that each molecule belongs when they were compared with a group of 211 synthetic PPARγ partial agonists. The 2D structures of the molecules of this table can be found in Figure S1.

- The genus name *Salvia* derives from the Latin *salvere* meaning “to save” perhaps referring to the healing properties of plants from this genus. Leafs, roots or flowers from species of *Salvia*, like *Salvia officinalis* [20], *Salvia miltiorrhiza* (a regional Chinese variety) [21], *Salvia fruticosa* [22] and *Salvia lavandulifolia* [23] have been used traditionally worldwide to treat diabetes [1]. The molecules deoxyneocryptotanshinone and miltionone I, which are found in *S. miltiorrhiza* extracts (Table 2), were predicted by our VS as PPARγ partial agonists, and they are extremely similar to the main lipophilic diterpene compounds from Danshen (*i.e.*, the dried root of *S. miltiorrhiza*), and in particular to tanshinone IIA (see Figure 1A for a comparison of the three structures). Tanshinone IIA enhances low-dose insulin-mediated tyrosine auto-phosphorylation of the insulin receptor β-subunit [24]. Although *S. miltiorrhiza* extracts have been shown to have anti-atherosclerotic and antidiabetic properties [21], there is not any evidence that relates the antidiabetic action of the extracts from *S. miltiorrhiza* with PPARγ. However, it is known that extracts from the leaves of *S. officinalis* activate PPARγ [6]. Deoxyneocryptotanshinone and miltionone I molecules may be useful for the development of a new class of specific insulin receptor activators that combine this action with the action of PPARγ partial agonists. In addition, we have predicted as PPARγ partial agonists three extra molecules from other extracts of *Salvia* whose species have never been described as antidiabetic: (a) sanigerone from *Salvia lanigera*; (b) 12-hydroxysapriparaquinone from *Salvia prionitis* and *Salvia eriophora* and (c) prionitin from *S. prionitis* (Table 3). These molecules are new candidates of PPARγ partial agonists.
- The 2′,5,6′-trihydroxy-6,7,8-trimethoxyflavone that is isolated from the roots of four species of plants of the genus *Scutellaria* (*Scutellaria baicalensis*, *Scutellaria adenostegia*, *Scutellaria alpina* and *Scutellaria ramosissima*), was also identified as a PPARγ partial agonist in our VS procedure (Table 2). Extracts from *S. baicalensis* are prescribed in Kampo medicines in Japan [1], and they are reported to enhance the antidiabetic activity of metformin [25]. Baicalein (5,6,7-trihydroxyflavone), a related compound of the flavone hit, isolated from the roots of *S. baicalensis* (see Figure 1B for a comparison of both structures), is an α-glucosidase inhibitor [26]. *S. baicalensis* extracts may therefore contain more than one active component with different modes of antidiabetic action.
- *Cryptolepis sanguinolenta*, a shrub indigenous to West Africa, has been employed by traditional healers in the treatment of various fevers, including malaria [27]. Cryptolepine, an indoloquinolone alkaloid isolated from *C. sanguinolenta*, significantly lowers glucose when given orally in a mouse model of diabetes [5], and its antihyperglycemic activity has been demonstrated by several cryptolepine analogs [28]. Cryptolepicarboline is a cryptolepine analog isolated from *C. sanguinolenta* [27] (see Figure 1C for a comparison of both structures) that we predict as a PPARγ partial agonist (see Table 2). This result suggests that the increase of glucose uptake caused by cryptolepine and analogous compounds could therefore be mediated by the action of PPARγ.
- Dysidine is a sesquiterpene quinone from the marine sponge *Dysidea villosa* that greatly promotes glucose uptake in 3T3-L1 cells and shows strong insulin-sensitizing activity [29]. The results of our VS procedure suggest that an analog of dysidine isolated from *D. villosa* (see Figure 1D for a comparison of both structures) may be a PPARγ partial agonist (Table 2). Although it has been suggested that dysidine exhibits its cellular effects through the activation of the insulin pathway, possibly through the inhibition of protein tyrosine phosphatases [29], it is possible that the mode of action of dysidine and analogous molecules could also be through the action of PPARγ, or that different components of a *D. villosa* extract show antidiabetic activity through different mechanisms. Dysidine and analogous molecules are therefore potential lead compounds for the discovery of new antidiabetic compounds.
- Xanthoangelol F from the Japanese plant *Angelica keiskei* significantly enhances glucose uptake without activating the transactivation activity of PPARγ [30]. This agrees with the results of our VS workflow that suggest that xanthoangelol F acts as a PPARγ partial agonist (Table 2). This compound may therefore belong to the interesting group of PPARγ partial agonists that stimulate glucose uptake without promoting the transactivation activity of PPARγ and avoid some of the problematic side effects of PPARγ full agonists [31], [32].
- The remaining 6 molecules predicted as PPARγ partial agonists through our VS workflow that belong to extracts with described antidiabetic properties (see Table 2) are: (a) 7-hydroxy-3,5,8-trimethoxyflavone from *Achyrocline satureoides*m, a species widely used as medicinal plant in South America; (b) 5-hydroxy-7,8,2′,3′-tetramethoxyflavone from *Andrographis paniculata*; (c) 2,4,6,2′,6′-pentamethoxybiphenyl isolated from *Fucus vesiculosus*; (d) hericerin from *Hericium erinaceum*; (e) the molecule with CAS number 78279-81-9 from *Aspergillus terreus*; and (f) bazouanthrone from *Harungana madagascariensis*. Our results suggest that these molecules could be PPARγ partial agonists and that extracts containing these molecules could stimulate glucose uptake through the action of PPARγ. This information is novel and relevant because it is the first time that antidiabetic properties for these molecules have been suggested.

**Table 3:** Natural extracts that contain one molecule predicted to be a PPARγ partial agonist by our VS protocol and that are related to natural extracts that are described to have antidiabetic activity.

Molecule Name (CAS number)	Cluster	Extract	Kingdom - Family	Ref. Isolation Molecule from Extract	Antidiabetic Extract	Ref. Antidiabetic Extract	Ref. Antidiabetic Molecule
7-hydroxydehydrothalicsimidine (218629-64-2)	12	*Annona purpurea*	Plantae - Annonaceae	[70]	*Annona squamosa*	[71]	-
artocarpin (7608-44-8)	7	*Artocarpus gomezianus*	Plantae - Moraceae	[72]	*Artocarpus heterophyllus*	[73]	-
6-O-Desmethylauricepyron (75680-08-9)	4	*Helichrysum stenopterum, H. odoratissimum, H. mixtum*	Plantae - Asteraceae	[74], [76]	*Helichrysum plicatum, H. graveolens*	[75]	-
1-(5,7-dimethoxy-2,2-dimethyl-2H-1-benzopyran-8-yl)-ethanone (31367-55-2)	5	*Melicope ptelefolia, M. simplex*	Plantae -Rutaceae	[77], [79]	*Evodia officinalis*	[78]	-
omphalocarpin (120693-45-0)	9	*Murraya paniculata*	Plantae - Rutaceae	[80]	*Murraya koeingii*	[4]	-
sanigerone (586960-68-1)	2	*Salvia lanigera*	Plantae - Lamiaceae	[81]	*Salvia lavandulifolia*	[23]	-
sapriparaquinone (119139-54-7)	2	*Salvia prionitis, S. eriophora*	Plantae - Lamiaceae	[82], [83]	*Salvia officinalis*	[20]	-
prionitin (117469-56-4)	11	*Salvia prionitis*	Plantae - Lamiaceae	[84]	*Salvia fruticosa*	[22]	-
1,3,5,8-tetramethoxy-9H-xanthen-9-one (54954-13-1)	8	*Swertia hookeri*	Plantae - Gentianaceae	[85]	*Swertia punicea, S. japonica, S. chirayita, S. paniculata*	[33], [34], [35], [87]	[33], [86]
nitenin (92590-02-8)	10	*Tephrosia watsoniana*	Plantae - Fabaceae	[88]	*Tephrosia purpurea*	[89]	-

The Table shows the natural extracts (*i.e.*, third column) and the VS hits that have been purified from them (identified by their common name, when available, and CAS number) and that are the related to extracts with described antidiabetic activity (*i.e.*, sixth column). The bibliographic references for each extract are split in three columns where (a) the fifth column reports papers that describe the purification of each molecule from the corresponding extract; (b) the seventh column reports papers that describe the antidiabetic activity of the related extract (see sixth column); and (c) the eighth column reports papers that describe the antidiabetic activity of the corresponding or similar molecules (when available). The second column represents the number of the cluster to which each molecule belongs when they were compared with a group of 211 synthetic PPARγ partial agonists. The 2D structures of the molecules of this table can be found in Figure S1.

Taking into account the fact that extracts from closer species of the same genus may share a high number of components, we also look for species that contain a molecule that we predict as a PPARγ partial agonist and, although they have been not described previously as antidiabetic, they are related (*i.e.*, they belong to the same genus) to species with known antidiabetic properties. Thus, we identified 10 molecules isolated from 16 different plants, such as *Acradenia franklinii*, *Annona purpurea*, *Artocarpus gomezianus*, *Euodia lunuankenda*, *Evodia elleryana*, *Helichrysum mixtum*, *Helichrysum odoratissimum*, *Helichrysum stenopterum*, *Melicope ptelefolia*, *Melicope simplex*, *Murraya paniculata*, *Salvia eriophora*, *Salvia lanigera*, *Salvia prionitis*, *Swertia hookeri* and *Tephrosia watsoniana* (Table 3 and Figure S1), whose extracts could show antidiabetic properties mediated by the action of PPARγ. For example, the 1,3,5,8-tetramethoxy-9H-xanthen-9-one from *Swertia hookeri* was identified as a PPARγ partial agonist by our VS (see Table 3). However, neither the molecule nor an extract from this species has been identified previously as an antidiabetic agent. Nevertheless, the whole plants of *Swertia japonica* and *Swertia chirayita* have been reported to exhibit hypoglycemic effects by oral administration, and the xanthone constituents, bellidifolin and methylswertianin, have been isolated as active constituents [33]–[35]. Methylswertianin and bellidifolin are molecules highly similar to the tetramethoxyxanthon from *Swertia hookeri* that we identified as a PPARγ partial agonist (see Figure 1E for a comparison of their structures). Our results therefore suggest that the antidiabetic action of *Swertia* species could be mediated at least in part by PPARγ.

To compare the 22 molecules from Tables 2 and 3 that we predict to be PPARγ partial agonists with known PPARγ partial agonists, we merged their structures with 211 structures of known PPARγ partial agonists obtained from the literature. The resulting set was classified into 26 clusters according to structure similarity. The 22 NP hits of our VS were classified into 12 clusters. None of these clusters contained any of the 211 known PPARγ partial agonists. Thus, our 22 predicted PPARγ partial agonists represent 12 different chemical scaffolds that are different from the ones present in known synthetic PPARγ partial agonists. Therefore, these scaffolds are lead-hoping candidates for searching for new PPARγ partial agonists.

### Conclusions

We have applied an experimentally validated VS workflow based on (a) two structure-based pharmacophores, (b) protein-ligand docking and (c) an electrostatic/shape similarity analysis to identify NPs that may be novel scaffolds for the discovery of new PPARγ partial agonists. Thus, from an initial set of 29,779 NPs that are annotated with their natural source, we predict 22 molecules to be potential PPARγ partial agonists. A subset of 12 of these molecules are present in 11 natural extracts with known antidiabetic activity and 10 of them are present in extracts related (*i.e.*, they are from species of the same genus) to plants with known antidiabetic activity. None of the 22 hits show chemical similarity with 211 known PPARγ partial agonists obtained from the literature and, therefore, are new chemical scaffold candidates for the development of PPARγ partial agonists. Moreover, our results provide a new hypothesis about the active molecules of natural extracts with antidiabetic properties and their mode of action, *i.e.*, the insulin-stimulated glucose uptake is increased through the action of PPARγ. We also suggest plants with undescribed antidiabetic activity that may contain PPARγ partial agonists and are related to plants with known antidiabetic activity. These plants represent a new source of potential antidiabetic extracts. Consequently, our work opens the door to the discovery of new antidiabetic extracts and molecules that can be of use, for instance, in the design of new antidiabetic drugs or functional foods focused towards the prevention/treatment of T2DM.

## Materials and Methods

### Initial Dataset of Natural Compounds Used

The initial in-house dataset of natural compounds that was filtered through the VS contained 29,779 compounds annotated with the natural sources from which they were obtained and the bibliographic references that describe how to extract them from each natural source. Moreover, according to the FAF-Drugs2 program [36], all of these molecules (a) show good ADME properties according to the Lipinski rule of five [37] (*i.e.*, only one violation of this rule was allowed) and (b) are not potentially toxic (*i.e.*, they lack “warhead” chelators, frequent hitters, promiscuous inhibitors and other undesirable functional groups). Conformations and sites for the 3D structures of these 29,779 compounds were determined during the generation of the corresponding Phase v3.1 (Schrödinger LLC., Portland, USA; http://www.schrodinger.com) [38] databases with the *Generate Phase Database* graphic front-end. Conformers are generated using the ConfGen facility. ConfGen carefully and systematically selects which conformations to produce, based upon an examination of the structure of the ligand being processed. During conformation generation, the ligand is first divided into a core region and a periphery. The conformational search generates all core configurations and then varies the peripheral configurations. The parameter values used during this conformer generation were the default values (*i.e.*, *Rapid* conformational sampling method and energy threshold of 25 kcal/mol for discarding conformers), with the exception of the maximum number of conformers per structure, which was increased from 100 (the default value) to 200. The conformer sites were generated with definitions made by adding the ability to consider aromatic rings as hydrophobic groups to the default built-in Phase definitions.

### Virtual Screening Workflow

The VS workflow used in this work is a slightly modified version of a VS workflow developed previously (that was also validated experimentally) to identify PPARγ partial agonists in chemical databases [18]. Briefly, the VS workflow consisted of several steps that must be applied sequentially (*i.e.*, the output molecules of one step were the input molecules for the next step). Thus, the filters applied (and sorted according their usage) were the following: (1) a structure-based antipharmacophore screening; (2) a structure-based pharmacophore screening (called partial agonist pharmacophore); and (3) an electrostatic/shape similarity analysis (the previously developed VS workflow was altered for the current work with lower threshold values for the electrostatic and shape comparisons; see below for more details). We have previously used a similar VS workflow to identify novel IKK-2 inhibitors [39], [40] and DPP-IV inhibitors [41], [42]. All of the PDB files used in that work were superposed with the DeepView v3.7 program (http://spdbv.vital-it.ch/) [43] to ensure that all of them had the same relative orientation. From then on, only the resulting re-oriented coordinates for these PDB files were used during the subsequent structure-based pharmacophore generation and in the steps of the VS workflow where spatial orientation is crucial (*i.e.*, pharmacophore-based searches, protein-ligand docking studies and shape and electrostatic-potential comparisons).

The initial set of compounds was filtered by a structure-based antipharmacophore with the aim of discarding potential PPARγ full agonists. This pharmacophore is formed by 5 sites (two hydrogen-bond acceptors and three hydrophobic sites) that are present in most of the validated 19 complexes of full agonists (where *validated* means that the coordinates for the ligand and the PPARγ active site are reliable according to their corresponding electron density map) and is completed with receptor-based excluded volumes obtained from the PDB file coded as 1FM9. Thus, this filter removed from the sample those molecules that had at least one *in vacuo*-generated conformer that matched at least 4 out of 5 sites of the antipharmacophore. The fitting between the molecules and the pharmacophore was analyzed with Phase v3.1 [38]. The subset of molecules that did not match the antipharmacophore was then used to identify possible partial agonists. To accomplish this task, a second pharmacophore obtained from the common sites of 12 validated complexes between PPARγ, and a partial agonist was used. It consists of one hydrogen-bond acceptor and three hydrophobic sites with receptor-based excluded volumes obtained from the PDB file coded as 2Q5S. Molecules that had at least one *in vacuo*-generated conformer and that matched with the 4 sites of the partial agonist pharmacophore were initially identified as putative PPARγ partial agonists. To find docking poses that were compatible with the partial agonist pharmacophore, those molecules identified as putative PPARγ partial agonists were then docked (using a rigid protein and flexible ligand) to the ligand-binding site of 2Q5S (ligands and water molecules from this structure were removed prior to docking). Thus, the best 32 docked poses predicted by the eHiTS v2009 program (SimBioSys Inc., Toronto, Canada; http://www.simbiosys.ca/ehits) [44] were filtered again with Phase through the partial agonist pharmacophore, using the same filtering options as the first pharmacophore matching, except that no re-orientation of the poses was allowed during the search. Default docking conditions were selected with the exception of the size of the sides of the cubic box encompassing the PPARγ binding site, which was increased from 10 to 15 Å. By default, eHiTS systematically evaluates all possible protonation states for the receptor and ligand, automatically for every ligand pose. Then during the docking algorithm each state is evaluated and scored and the most favorable state is selected [44].

The aligned poses that passed the pharmacophore and docking screenings were submitted to an electrostatic/shape similarity analysis, using the PPARγ partial agonists crystallized in the structures 2G0H, 4PRG, 2Q5S, 2FVJ and 2Q6S as a queries. Both electrostatic and shape similarity analyses was performed with EON v2.0.1 (OpenEye Scientific Software, Inc., Santa Fe, New Mexico, USA; http://www.eyesopen.com). EON is an electrostatics comparison program that compares electrostatic potential maps of pre-aligned molecules and determines the Electrostatic Tanimoto combo (ET_combo) score as the similarity criteria. In our case, the molecules were pre-aligned at the pharmacophore and docking steps. As the electrostatic potential of molecules is not strongly linked to the molecular graph, EON can screen molecular databases for electrostatic similarity to a query compound. The ET_combo score is the sum of two calculations: (a) the Shape Tanimoto (ST) score, which is a quantitative measure of three-dimensional overlap (where 1 corresponds to a perfect overlap, *i.e.*, the same shape) and (b) the Possion-Boltzman Electrostatic Tanimoto (ET_pb) score that compares the electrostatic potential of two small molecules and ranges from 1 (identical potential) to negative values that results from the overlap of positive and negative charges. In this work, we selected the EON thresholds taking into account the results of the comparison between a group of experimental poses for PPARγ partial agonists in their complexes with PPARγ. Applying the five query poses against twelve other experimental poses of previously described PPARγ partial agonists, the lowest values for the ET_pb score and ST were 0.2 and 0.4, respectively. Therefore, these values were used as thresholds during the VS electrostatic/shape similarity analysis. The thresholds used in the original VS workflow [18] were more restrictive (0.3 and 0.5 for ET_pb and ST, respectively).

### Virtual Screening Workflow Validation

The ability of the VS workflow to identify PPARγ partial agonists was tested by applying it to a group of 211 known PPARγ partial agonists obtained from the literature and 3,122 decoys obtained from the DUD database [19]. The structures of the 211 partial agonists were built with ChemDraw Ultra v11.0 (CambridgeSoft Corporation, Cambridge, MA, USA; http://www.cambridgesoft.com/) [45] and were cleaned using LigPrep v2.3 (Schrödinger LLC., Portland, USA; http://www.schrodinger.com). We calculated an **EF** and values for sensitivity (**Se**) and specificity (**Sp**) for the global VS workflow and each step [46]. The **EF** was obtained as the quotient between the fraction of actives in the sample that *survived* a particular VS step and the fraction of actives that were in the sample before applying this step. The **EF** therefore represents the ratio of the number of actives actually retrieved by a method compared to the number expected purely by chance. **Se** describes how well the model correctly identifies active compounds and it is calculated as the ratio between the number of active molecules that *survived* a particular VS step and the number of all active compounds that were in the sample before applying the VS step. **Sp** measures the correct assignment of inactive compounds. It is calculated as the ratio between the number of inactive compounds that were discarded at a particular VS step and the number of all inactive molecules that were in the sample before applying the VS step. Because the aim of the antipharmacophore step was to remove full agonists of PPARγ, for this step an **EF** and values of **Se** and **Sp** could not be estimated.

### Structural Similarity Analysis

To obtain new scaffolds for PPARγ partial agonists, the VS hits were merged with the 211 PPARγ partial agonists previously used for validating the VS workflow and clustered with Canvas v1.2 (Schrödinger LLC., Portland, USA; http://www.schrodinger.com). Using a fingerprint precision of 32 bits, MOLPRINT2D fingerprints [47] were calculated for each molecule, and then a hierarchical clustering, based on Tanimoto similarities, was obtained. The number of clusters obtained was defined using the Kelley criterion [48].

### Hardware specifications

We used an Intel Core 2 Quad Q8200 (2.33 GhZ) equipped with 4 GB RAM running Linux Ubuntu 10.04.

## Supporting Information

Figure S1 **2D-structures, product name and CAS number of the molecules predicted to be PPARγ partial agonists.**
(PDF)
