# Supplementary figures and images for: Identification of PPARgamma Partial Agonists of Natural Origin (II): In Silico Prediction in Natural Extracts with Known Antidiabetic Activity
Source: PLoS One. 2013 Feb 6;8(2):e55889. doi: 10.1371/journal.pone.0055889 (PMC3566095; doi:10.1371/journal.pone.0055889)

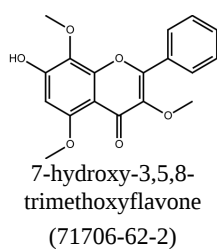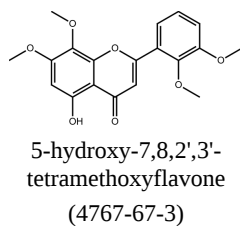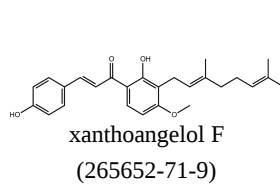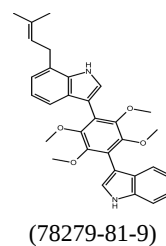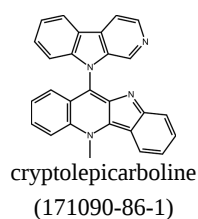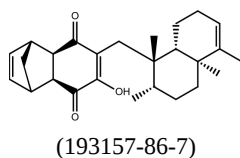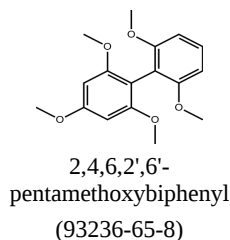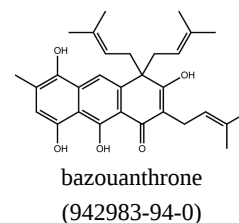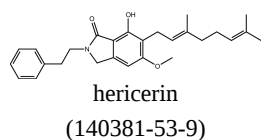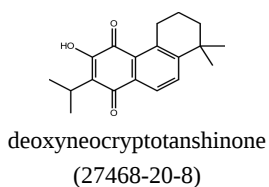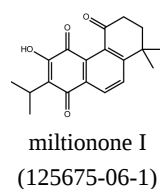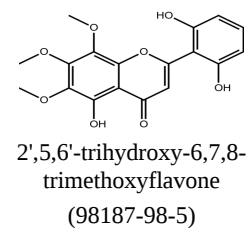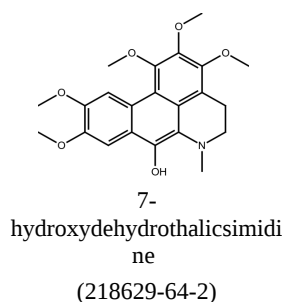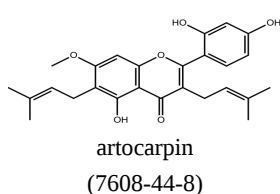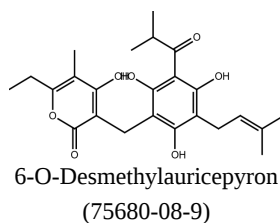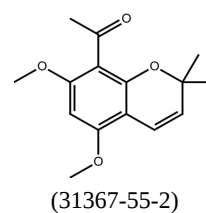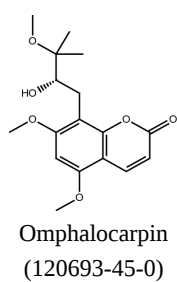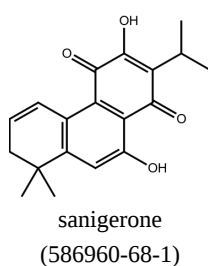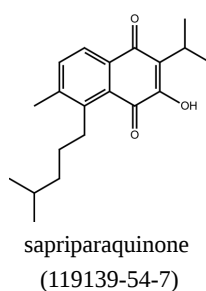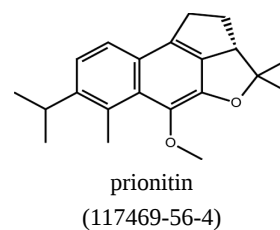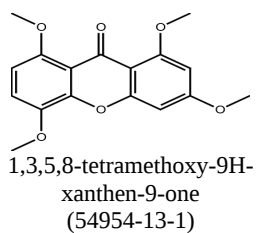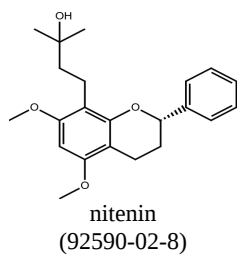

Supplement: Figure S1 — 2D-structures, product name and CAS number of the molecules predicted to be PPARγ partial agonists. (PDF) [file pone.0055889.s001.pdf]
